# When hollow multishelled structures (HoMSs) meet metal–organic frameworks (MOFs)

**DOI:** 10.1039/d0sc01284j

**Published:** 2020-04-30

**Authors:** Zumin Wang, Nailiang Yang, Dan Wang

**Affiliations:** State Key Laboratory of Biochemical Engineering, Institute of Process Engineering, Chinese Academy of Sciences 1 North 2nd Street, Zhongguancun, Haidian District Beijing 100190 China danwang@ipe.ac.cn; University of Chinese Academy of Sciences 19A Yuquan Road Beijing 100049 P. R. China

## Abstract

Hollow multishelled structures (HoMSs) have distinguished advantages, such as a large effective surface area, an optimized mass transport route, and a high loading capacity, but the fabrication of HoMSs has been a big challenge. In 2009, we developed a universal and facile method for HoMS fabrication, *i.e.*, the sequential templating approach (STA). Progress in the synthetic methodology has enabled the study of HoMSs to develop and has made it a research hotspot in materials science. To date, HoMSs have shown their advantages in a wide range of applications, including catalysis, energy conversion and storage, drug delivery, *etc.* Based on the understanding in this field, we recently revealed the unique temporal–spatial ordering properties of HoMSs. Furthermore, we have been wondering if the structure of a HoMS can be modulated at the molecular level. Encouragingly, metal–organic frameworks (MOFs) are star materials with clearly defined molecular structures. The compositions, geometries, functionalities and topologies of MOFs have been well tuned by rational design. Integrating the unique properties of MOFs and HoMS could realize the systemic design of materials from the molecular to the micro-level, which would provide a series of advantages for various applications, such as developing high performance catalysts for cascade and/or selective catalysis, combining the reaction and separation process for multiple reactions, releasing drugs in a certain environment for smart medicine, and so on. We believe it is time to summarize the recent progress in the integration of MOFs and HoMSs, including HoMSs coated with MOFs, MOF-derived HoMSs, and MOFs with a hollow multishelled structure, and we also put forward our personal outlook in relation to the future opportunities and challenges in this emerging yet promising research field.

## Abstract 2

Three major kinds of integrations of MOFs and HoMSs and possible further applications are discussed.

## Graphical abstract

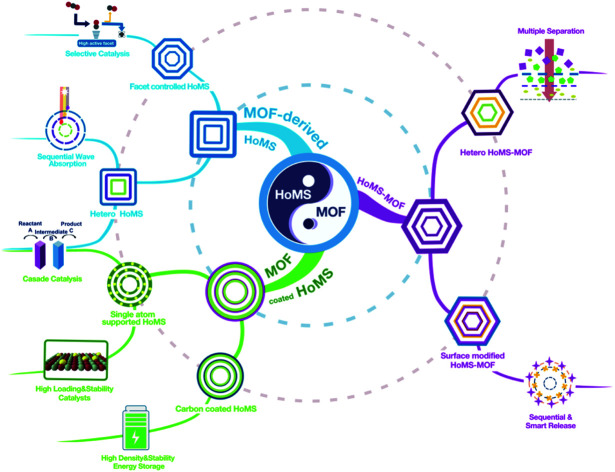

## 1. Introduction

Hollow multishelled structures (HoMSs) are a major type of burgeoning advanced nanostructure which have at least two shells with corresponding internal cavities, and the multiple shells are arranged in order from outside to inside.^1^ In contrast to the random aggregation of nanoparticles or a single-shelled hollow structure, HoMSs have the irreplaceable advantages arising from a temporal–spatial ordered structure.^2^ More specifically, materials with well-separated shells can perform different functionalities within one small micro–nano system. More importantly, the physical/chemical processes take place on or through each shell sequentially with specific orders from outside to inside or in reverse.^2^ This is crucial for applications such as selective cascade reactions, smart drug release, stepwise separation and sequential light harvesting.^3^ Since 2009, our group has developed a universal and facile method for the synthesis of HoMSs, namely the sequential templating approach (STA). By precisely controlling the reaction thermodynamics and kinetics, the micro–nano structures of HoMS can been controlled.^4–8^ Afterwards, this breakthrough in synthetic methodology made HoMS a hotspot in material science, and various related applications have been rapidly developed.^7,9–14^ HoMS material has been widely recognized as one of the most promising functional materials.^15^ However, the precise fabrication of HoMSs (with the composition of metal oxide/sulfide/phosphide, not the MOF-based HoMS, unless otherwise specified) on the molecular level is still confronted with challenges. The micro-level HoMS particle is assembled by nanoparticles, which leads to irregular channel structure and broad pore size distribution, thus bringing about difficulties in the study of mass transport in the HoMSs. Also, the exposed facet of these nanoparticles was still difficult to control until our recent report using a topological chemical reaction to convert metal organic frameworks (MOFs) into HoMSs.^16^ A MOF is a kind of porous crystalline material formed by the linkage of metal ions or clusters with organic ligands.^17–20^ The topologically ordered crystalline structure endows MOFs with the advantages of a uniform and adjustable pore size, an ordered atom arrangement, and a tailorable functionality, making them promising in a wide range of applications.^21–25^

**Scheme 1:**
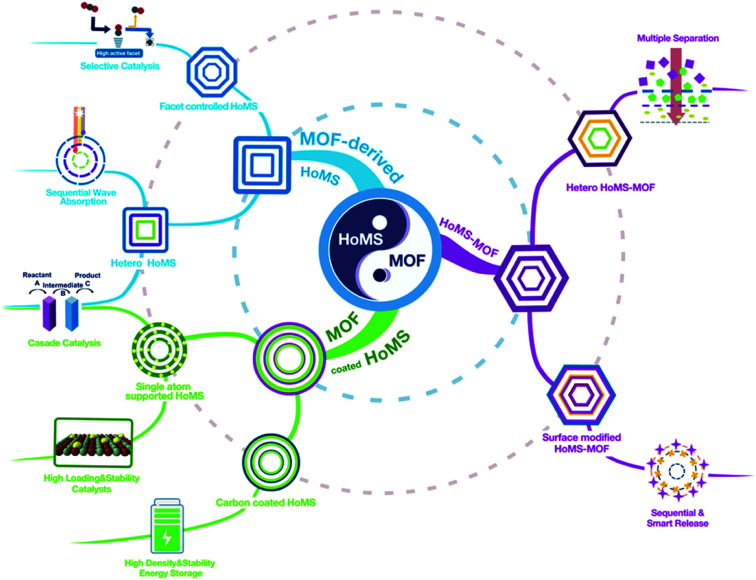
The integration of a MOF and a HoMS. (1) The MOF can be coated on the surface of the HoMS and it can be further converted into single atoms and carbon materials (green route). (2) MOF-derived HoMSs (blue route) and (3) HoMS–MOF (purple route) are also important and can have different compositions/textures/facets/functional groups in each shell. All of the conversions can combine the advantages of the HoMS and the MOF and bring about lots of applications in energy, environment, catalysis and biology fields.

In this case, it is of conceptual novelty to integrate MOFs with HoMSs, which may bring more possibilities to guide material design and exploit new applications. Firstly, the specific properties of the coordinated metal atom arrangement and the ordered pores in the MOFs can endow the derived HoMS material with a controllable micro/nano structure. Secondly, in reverse, fabricating MOFs with HoMSs can endow the MOFs with temporal–spatial ordering properties, optimized mass transfer and abundant and accessible reaction sites, extending their applications. Thirdly, the rational design of heterogeneous HoMSs with MOFs can provide synergistic effects for a certain reaction. Note that the speciality of MOF-based HoMSs is the cavity between the shells, which is quite different from the compact MOF-based core–shell structure. These cavities can perform as a micro reactor to enrich the substrate, raise the partial pressure, promote diffusion, and further accelerate the reaction. Meanwhile, this unique hollow multishelled structure can provide a larger effective surface area for reactions. Thus, the merits from both the material (MOF) side and the structure (HoMS) side result in a perfect combination to realise properties that could not be achieved elsewhere. Excellent work from all around the world has attracted enormous attention from diverse disciplines. In this case, it is about time to survey the progress of related research, exploit the fabrication approach for the combination of MOFs and HoMSs, discuss the structure-induced properties, and furthermore to provide new perspectives and directions to further increase the development of this field. Herein, this perspective article systematically covers the correlations between MOFs and HoMSs, it is divided into three parts, including coating HoMSs with MOFs, deriving HoMSs from MOFs, and fabricating hollow multi-shelled MOFs. At the end, we also put forward our personal outlook of the future opportunities and challenges in this promising research field (Scheme 1).

## 2. HoMSs with MOF coatings

**Fig. 1:**
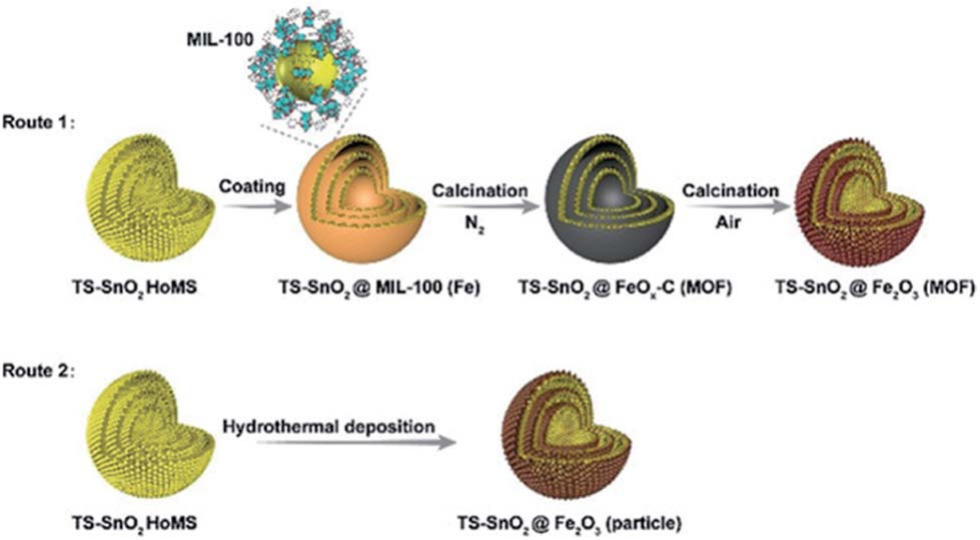
Synthesis procedure to produce HoMSs with MOF-derived casings involving two calcination steps (Route 1) or a hydrothermal deposition method (Route 2). Reproduced with permission.^29^ Copyright 2019, Wiley-VCH.

The most straightforward and rational approach to hybridise the advantages of HoMSs and MOFs is to coat MOFs on HoMSs to produce MOF-coated HoMS. Because MOFs have a good affinity to various substrates^26–28^ it is not a big challenge to fabricate MOF-coated HoMS hybrids experimentally. The first paper in this topic is published in 2019 by Wang's group.^29^ Take the synthesis of HoMSs SnO_2_@MIL-100(Fe) as an example, triple-shelled SnO_2_ hollow structures were synthesized first with STA, and then the SnO_2_ HoMSs were modified with PVP, which was conducive to capturing MOF precursors for heterogeneous nucleation. After soaking the HoMSs in Fe^3+^ solution and H_3_BTC solution alternately several times, a dense MOF coating could be uniformly formed on the SnO_2_ HoMSs (Fig. 1), and the thickness of the MOF shells could be easily controlled by adjusting the number of repeated cycles. This method is also universal and can be extended to the casing of other HoMSs (*e.g.* TiO_2_, NiO, Co_3_O_4_).

Furthermore, a precise post-treatment can transform the coated MOF into porous carbons,^30^ metal oxides,^31^ metal active centres^32^ or single atoms,^33^ but can retain the structural information of the MOFs and demonstrate a positive effect on the performance of the substrate materials for various applications. For instance, the aforementioned MOF-coated HoMS, *i.e.*, SnO_2_@MIL-100(Fe), was post-treated by a two-step calcination, *i.e.*, first with N_2_ protection and then in air. By precisely tailoring the pyrolysis process of the MOFs, HoMSs in heterogeneous casings, such as SnO_2_@Fe_2_O_3_ (MOF)–HoMS and SnO_2_@FeO_*x*_–C (MOF)–HoMS, were obtained. The characteristics of the large surface areas and porous structures of MOFs can be largely retained in their derived casings. The specific surface area of SnO_2_@Fe_2_O_3_(MOF)–HoMS was eight times larger than that of pure SnO_2_ HoMS, and SnO_2_@Fe_2_O_3_(MOF)–HoMS also had an increased pore volume and porosity. As a consequence, the SnO_2_@Fe_2_O_3_(MOF)–HoMS hybrid worked as a superior anode material in lithium ion batteries, showing a higher specific surface area and more lithium-storage sites, and thus a larger storage capacity could be provided. It also demonstrated excellent structural stability, providing promoted reversibility of the active materials. The cycling ability improved dramatically. The retention capacity after 100 cycles of the SnO_2_@Fe_2_O_3_ (MOF)–HoMS increased eightfold compared with SnO_2_@Fe_2_O_3_(particle)–HoMS, which demonstrated the superiority of the casing originated from the MOFs. The porous hierarchical structure in SnO_2_@Fe_2_O_3_ (MOF)–HoMS also facilitated the ion diffusion and decreased the charge resistance, leading to the significantly improved rate performance. Specifically, the capacity of SnO_2_@Fe_2_O_3_(MOF)–HoMS was triple that of the SnO_2_ HoMSs at the same current density of 2 A g^−1^ because of the rapid charge/ion transfer within the porous and “breathable” MOF-derived casing.^29^

Moreover, the hybrid can also provide more space to store the reactants, which benefits reactant accumulation in the local environment.^34^ For instance, Prof. Yaghi discovered that although a MOF is not active for gas-phase hydrogenative conversion of methyl-cyclopentane, it increases the activity by enhancing local concentrations of the reactant and H_2_ inside the MOF crystals and endows the catalyst with unusual selectivity *via* kinetic changes of the adsorption geometries of the transition states or intermediate species within the pores of the MOF.^35^ It can be predicted that hybridizing the advantages of HoMSs, such as efficient light utilization,^36^ abundant effective surface areas^37^ and fast mass transmission,^38^ with those of MOFs can further broaden their applications.

## 3. MOF-derived HoMSs

STA refers to the approach which starts from a template rich precursor, and then this template can direct the process sequentially multiple times during its removal. Widely used templates include metal-rich carbonaceous spheres^39^ or polymer particles,^40^ and metal-polymer hybrid particles.^41^ Among them, MOFs, with beautifully designed structures and metal-rich compositions, can be considered as an excellent template for STA. Compared with conventional templates, MOFs show several characteristics in the fabrication of HoMSs: (1) MOFs can work as both metal-rich precursors and templates, thus there is no necessity for further ion absorption process; (2) the metal motif dispersion can be considered as relatively uniform within the MOF, without the concentration gradient; (3) the obtained materials normally resemble the shape of parent MOFs to get a variety of morphologies; (4) the MOF-derived materials often have a well-defined hierarchical pore structure and an easily tailored composition. Above all, MOFs have been highlighted in recent years as unique precursors for HoMSs with design possibilities. Moreover, we would like to mention that the precise control of the reaction at the interface is significantly important for the formation of hollow materials with a certain micro/nano structure. Only the accurate control of the HoMS synthesis process can release all the structural potential of MOFs.

Calcination/annealing is the most common way to synthesize metal oxide HoMS through STA.^1^ During calcination, a temperature gradient along the radial direction was established. The outer parts of the MOFs firstly decomposed to form a rigid metal oxide shell while the inner part remained unpyrolyzed. Afterwards, the continuous mass loss and volume shrinkage led to the separation of the metal oxide shells from the inner MOF cores, and the inner MOF cores can act as the following templates to repeat this process several times, resulting in hollow structures with multiple shells.^42,43^ Moreover, because of the flexibility in the chemical composition and structure in the MOF, the composition of the HoMS can be easily tuned.^44,45^ As reported, metal oxide HoMSs with different compositions, doping elements and mixtures can be obtained. Especially, MOFs with a variety of morphologies can be readily prepared,^46–48^ thus HoMSs with distinctive morphologies other than spheres are expected to be fabricated through STA by using MOFs as templates. For example, Co/Mn-ZIFs dodecahedra were firstly obtained at room temperature by co-precipitating metal salts and a linker in methanol. After a continuous two-step calcination, manganese-cobalt oxide HoMSs with a dodecahedron shape were prepared, and the shell number could be controlled from single to triple by adjusting the calcination conditions.^42^

**Fig. 2:**
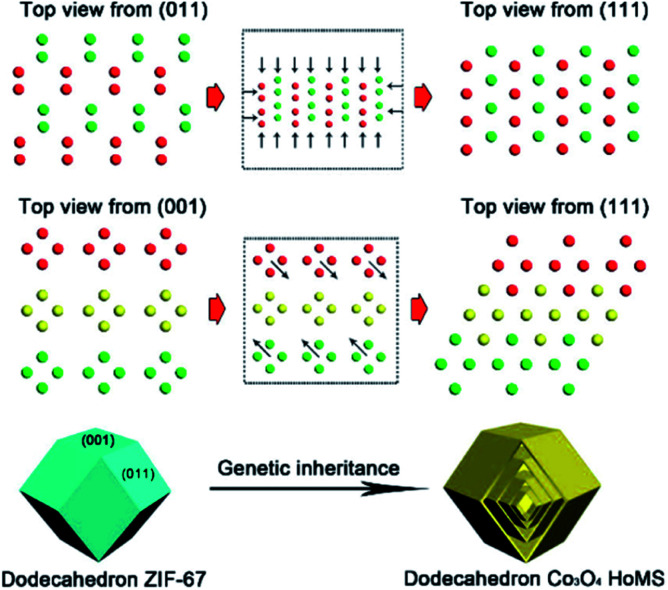
“Genetic inheritance” from the MOFs to the Co_3_O_4_ HoMSs. All the atoms are Co and the different colors (green, yellow, and red) indicate periodic units. Reproduced with permission.^16^ Copyright 2019, American Chemical Society.

The most significant feature of adopting MOFs as templates is using the topological structure of their atomic arrangement. The arranging pattern of atoms in MOFs can have an unignorable impact on the atom arrangement of the final product. By controlling the calcination parameters (such as oxygen partial pressure and heating rate), the surface crystalline structure of the obtained materials can be regulated. After deeply studying the Co atom arrangement in ZIF67, we noticed that the spatial distribution of the Co atoms within the exposed (001) and (011) facets of ZIF67 demonstrated an arrangement similar to the (111) facet of Co_3_O_4_. Hence, during thermal decomposition, Co atoms tended to topologically shift within the crystal lattice to form Co_3_O_4_ with the (111) facets exposed predominantly, instead of relocating through complicated rotation, torsion, and migration to expose other facets like (311), which is the dominant facet prepared by other methods. Because of this so-called topological transmission, the atoms transferred in desired directions to selectively expose the (111) crystal facets. Consequently, a triple-shelled Co_3_O_4_ hollow dodecahedron with a unique shell preferably exposing the (111) facets was prepared (Fig. 2). Considering the Co_3_O_4_ (111) facets have stronger interactions with CO_2_, the structure can possess higher activity towards photocatalytic CO_2_ reduction. In this case, owing to the facet control, the activity of the MOF-derived HoMSs is about 5- and 3-times higher than that of the Co_3_O_4_ nanoparticles (NPs) and Co_3_O_4_ hollow spheres prepared by carbon sphere templates, respectively.^16^

**Fig. 3:**
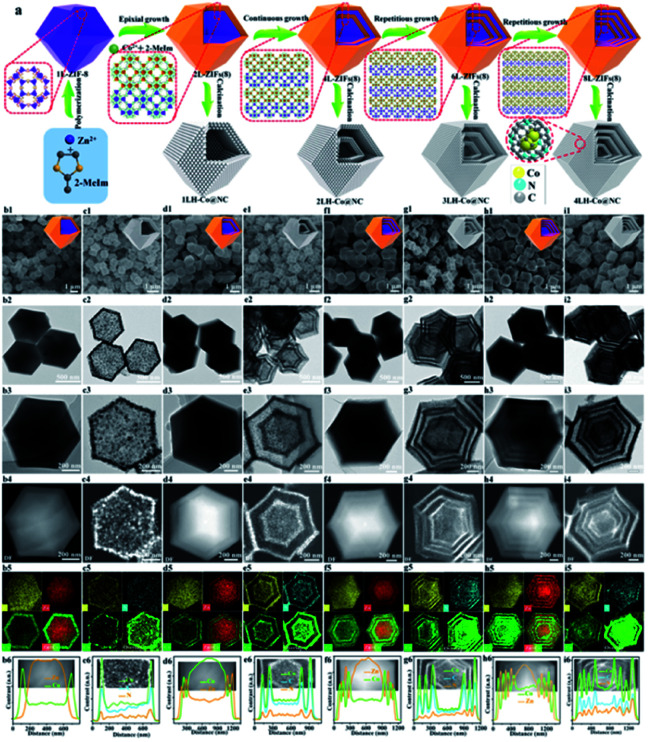
Controllable fabrication of multilayer ZIF67 and the derived solid, yolk–shell hollow, and multi-shell hollow Co@N doped carbon nanoarchitectures. (a) A schematic diagram of the synthesis. (b1–i1) SEM images, (b2–i2) TEM images, (b3–i3) HAADF-STEM and EDS mapping images, and (b4–i4) elemental line scan profiles. Reproduced with permission.^49^ Copyright 2019, American Chemical Society.

Another promising factor of different MOFs is the different graphitization degree during calcination, which can be considered as a unique method for HoMS fabrication. By using this knowledge, Zhang *et al.* firstly prepared five layered ZIF67@ZIF8@ZIF67@ZIF8@ZIF67 solid structures through shell-by-shell epitaxial growth. Then the obtained materials were heated in nitrogen atmosphere. During pyrolysis, the organic component in ZIF8 converted into amorphous carbon, while the organic component in ZIF67 converted into porous carbon with a higher degree of graphitization. Later on, the inner amorphous carbon underwent an inside–out Ostwald ripening process, diffused outward and further graphitized into dense shells, which was catalysed by the Co NPs derived from the exterior ZIF67 component, leaving interior voids between the shells. By controlling the shell-by-shell coating procedures, different multi-layered structures can be easily synthesized, resulting in different complex nitrogen-doped hollow porous carbon architectures, including multi-shell, yolk–shell, and yolk–multi-shell structures (Fig. 3). Owing to their hierarchical micro/mesoporous structure, high nitrogen doping, large surface area, graphitic structure, and the unique multi-shell hollow structure, the MOF-derived nitrogen-doped carbon HoMS dodecahedrons exhibited a distinguished capacitance of 346 F g^−1^ at a current density of 0.5 A g^−1^, excellent stability with approximately 93% capacitance retention after 10 000 cycles, and a high energy density of 11.64 W h kg^−1^ at a power density of 250 W kg^−1^ as a supercapacitor.^49^

Ion-exchange is also an effective way to prepare HoMSs.^50^ For instance, precisely controlling the interfacial reaction between the solid MOF and the solution (especially the reagent) can effectively convert MOFs into HoMS materials. For example, during the reflux process, the ZnS shell can be formed on the surface of the solid ZIF8 precursor. Then the inner core shrank, and the outer shell became thick. During quenching, the adhesive force from the outer shell was released and the fresh surface of the inner core ZIF8 was exposed. The yolk–shell particles with an inner core of ZIF8 and an outer shell of amorphous ZnS were formed. After performing another sulfidation process, another shell can be formed on the fresh surface of ZIF8. Hence, the ZnS HoMS could be obtained after three quenching and sulfidation processes. Afterward, the Sb_2_S_3_ HoMS can be achieved *via* a simple ion-exchange method by replacing Zn atoms with Sb^2+^ ions.^51^

Furthermore, HoMSs with more complex architectures can be achieved through controllable processing reaction steps. For example, Zhang *et al.* utilized two separate reactions to prepare unique double-shelled HoMSs with an inner shell of Co(OH)_2_ and an outer shell of NiCo-LDH. By depositing ZIF67 into an ethanol solution of Ni(NO_3_)_2_, LDH shells were formed on the outside of ZIF67 rhombic dodecahedra. Then, the single-shelled ZIF67@LDH was placed into Na_2_MoO_4_ aqueous solution to trigger the second reaction and fabricate double-shelled Co(OH)_2_@LDH hollow polyhedra.^52^ Hierarchical double-shelled hollow structures with different shell subunits can be obtained as well through delicate manipulation of the template-engaged reaction. Starting from ZIF67 nanocubes, Hu *et al.* managed the interfacial reaction with water to prepare ZIF67/Co(OH)_2_ yolk–shelled structures and further transformed them into a CoS double-shelled structure using a sequential reaction with Na_2_S. Interestingly, the inner CoS shell was composed of NPs, whereas the outer shell consisted of assembled nanosheets, in spite of the same composition.^53^

**Fig. 4:**
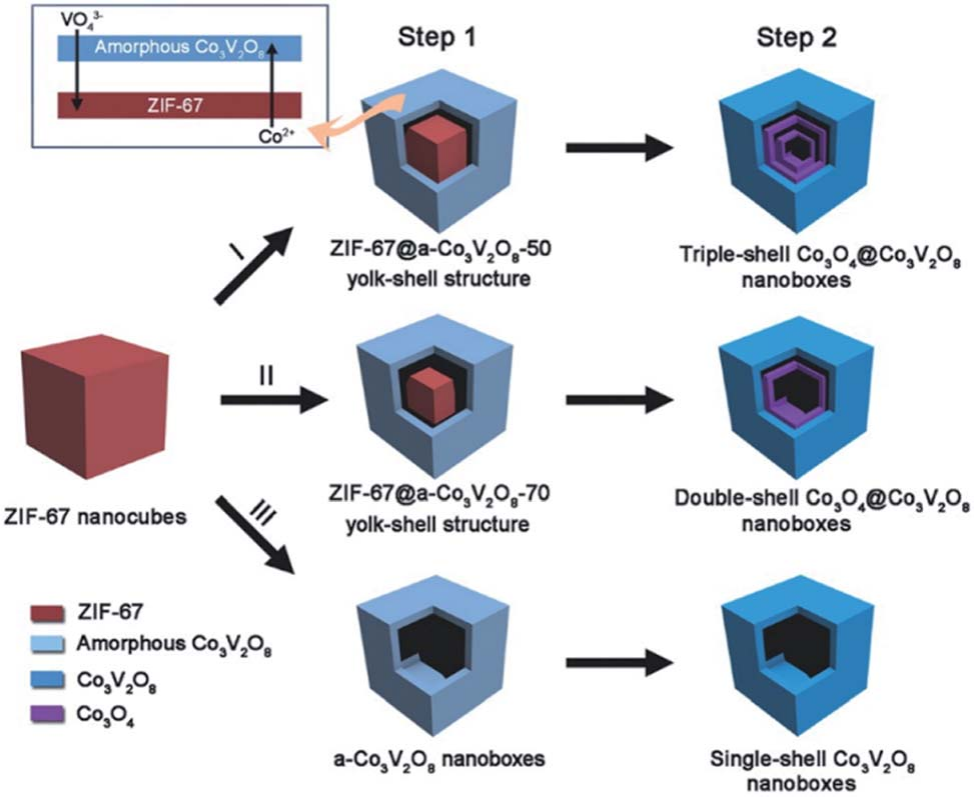
A schematic diagram of the formation of complex Co_3_O_4_@Co_3_V_2_O_8_ hollow structures. Step 1 involves solvothermal reactions with different amounts of vanadium oxytriisopropoxide (VOT): (I) 50 μL, (II) 70 μL, and (III) 120 μL. Step 2 is simple annealing in air. Reproduced with permission.^54^ Copyright 2018, Wiley-VCH.

It can be observed from the aforementioned examples that the precise reaction control at the interface (liquid/solid and gas/solid) is significantly important to direct the precise HoMS fabrication. In this case, by combining the interfacial reaction method with the calcination approach, complex HoMSs with a heterogeneous composition in different shells can be constructed. For example, Lu *et al.* prepared triple-shelled Co_3_O_4_@Co_3_V_2_O_8_ HoMS, in which the outermost shell was Co_3_V_2_O_8_ while the inner two shells were Co_3_O_4_.^54^ In detail, firstly, these ZIF67 nanocubes were dispersed in an absolute ethanol solution of vanadium oxytriisopropoxide (VOT) and continuously stirred for 20 min to form a clear solution. Then a ZIF67@amorphous-Co_3_V_2_O_8_ yolk–shell-structure was obtained after a solvothermal treatment of the aforementioned solution with a VOT concentration of 50 μL as shown in Step 1 (Fig. 4), during which, a vanadate anion (VO_4_^3−^) generated from VOT is considered to gradually replace the 2-methylimidazole anion in ZIF67 by ion exchange to form the amorphous-Co_3_V_2_O_8_ shell. Meanwhile, the continuous consumption of the ZIF67 core results in a gap space between the newly formed amorphous-Co_3_V_2_O_8_ shell and the remaining core. Afterward, a thermal calcination in air was applied to convert the amorphous-Co_3_V_2_O_8_ shell into a crystalline one and the ZIF67 core was converted into Co_3_O_4_ hollow shells. As such, triple-shelled Co_3_O_4_@Co_3_V_2_O_8_ nanoboxes can be eventually obtained. By precisely tuning the VOT concentration, two other hollow nanostructures, *i.e.*, double-shelled Co_3_O_4_@Co_3_V_2_O_8_ and single-shelled Co_3_V_2_O_8_ nanoboxes, can also be synthesized. The different compositions of the different shells can be easily observed from HR-TEM and EDS element mapping.

## 4. HoMS–MOFs

In reverse, MOFs with a hollow multi-shelled structure (HoMS–MOFs) can endow MOFs with the unique properties of HoMSs. As previously mentioned, MOFs are one of the most attractive materials with great promise in a variety of important applications, including adsorption,^55^ storage,^56^ separation,^57^ detection,^58^ photoluminescence,^59^ catalysis^60^ and biomedicine.^61^ Great efforts have been devoted to the design and preparation of MOFs with exquisite topologic structures to improve their performance or exploit new properties. Especially, the unique distinguished micro-nanostructural features of HoMSs are conducive to energy storage,^62^ absorption,^63^ sensing,^64^ catalysis,^65^ drug delivery,^66^*etc.* The fabrication of HoMS–MOFs may reinforce the usefulness of MOF materials and expand the scope of utilization of these materials.

**Fig. 5:**
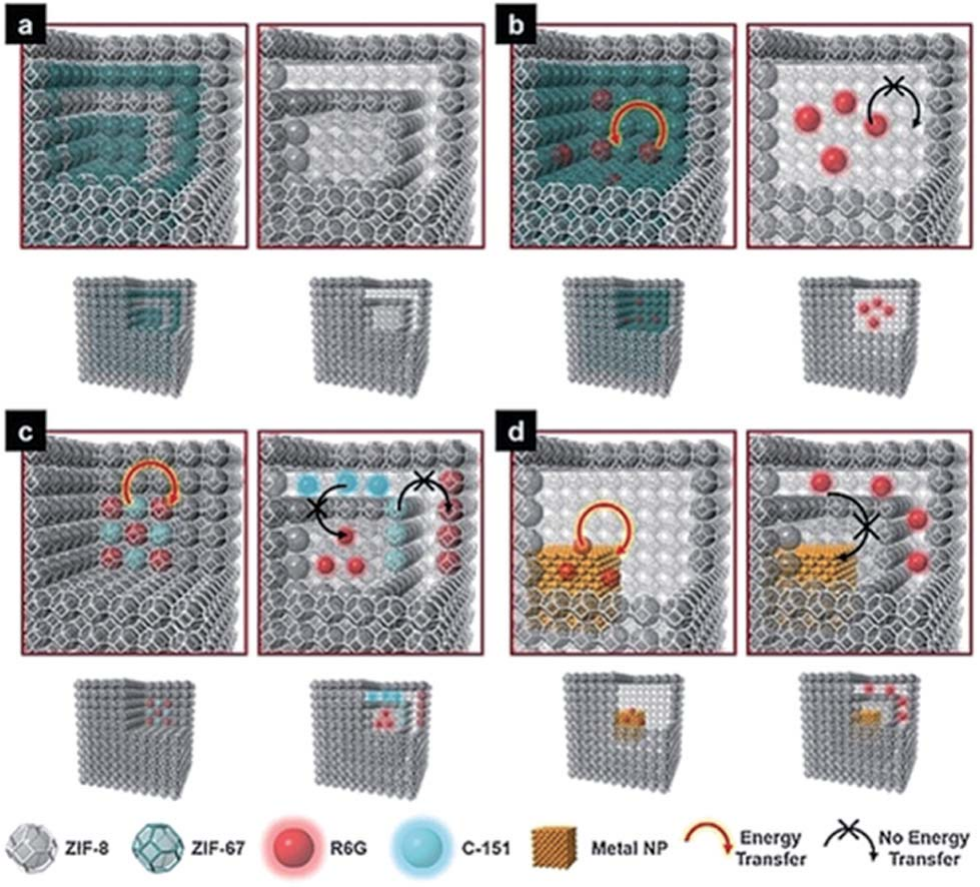
Models of multi-shelled ZIFs and the guest-to-host and guest-to-guest energy transfer. (a) Double-shelled ZIF67@ZIF8 (left) and double-shelled hollow ZIF8 (right); (b) energy transfer (ET) between guest R6G and host ZIF67 (left), and prohibited ET in hollow ZIF8 (right); (c) ET between guest R6G and guest C-151 (left), and prohibited ET between guests (right); and (d) ET between guest R6G and a guest Pd nanoparticle (left) and prohibited ET (right). Reproduced with permission.^67^ Copyright 2018, Wiley-VCH.

Hard-templating methods are the most conceptually straightforward route, and HoMS–MOFs can be achieved through shell-by-shell assembly and selective etching. The synthesis always requires multi-step coating alternately with target materials and interlayer materials, followed by selectively removing templates and interlayers according to the different stabilities.^67^ A typical example was demonstrated by Tsung's group. Because ZIF67 and ZIF8 share the same topology and crystal structure but have different metal nodes, they can epitaxially grow on each other easily. The onion-like multi-layered ZIF67@ZIF8 solid nanocubes can be prepared by shell-by-shell overgrowth. As ZIF67 was less stable than ZIF8 in the water/methanol solution, Co ions were released due to the dissolution of ZIF67, while the ZIF8 parts remained stable. When ZIF67 was completely removed, leaving cavities between the ZIF8 layers, the HoMS ZIF8 was obtained. Importantly, the multi-shelled MOFs can work as a novel host matrix to confine different kinds of guests at individual locations to mimic biological structures. More specifically, the guests originally immobilized in the ZIF8 layers are still there, while the guests originally immobilized in the ZIF67 layers are released and confined in the cavities between the ZIF8 shells. The chromophore molecules and the metal nanoparticles (NPs) can be precisely controlled either anchored on the MOF shells or in the cavities between the shells. In this case, Förster resonance energy transfer (FRET) molecules, rhodamine 6G (R6G) and 7-amino-4-(trifluoromethyl)coumarin (C-151) were selected as the molecule guests, and Pd NPs were selected as the heterogeneous guests. When the guests were fixed in the MOFs, the energy transfer was promoted; while if the guests were encapsulated in the cavities, the native flexibility of the surface functional groups can be reserved. The former is essential to applications related to energy transfer (ET) processes, like photo-fluorescence. As NPs and molecules usually provide different functions, the integration of them into one system with controlled ET is reasonable. If R6G molecules and Pd NPs were located in one cavity, the fluorescence would be largely reduced due to the quenching effect. However, when Pd NPs and R6G were separated into different cavities within the HoMS, enhanced fluorescence was detected because the quenching process was effectively inhibited by the separation of the ZIF8 shell. In this way, the mobility of the guests can be switched according to different application demands. Thus, the guest-to-host and guest-to-guest interactions can be regulated. This work enabled the precise separation control of multi-guests within the HoMSs, providing a new multifunctional platform (Fig. 5).^67^

**Fig. 6:**
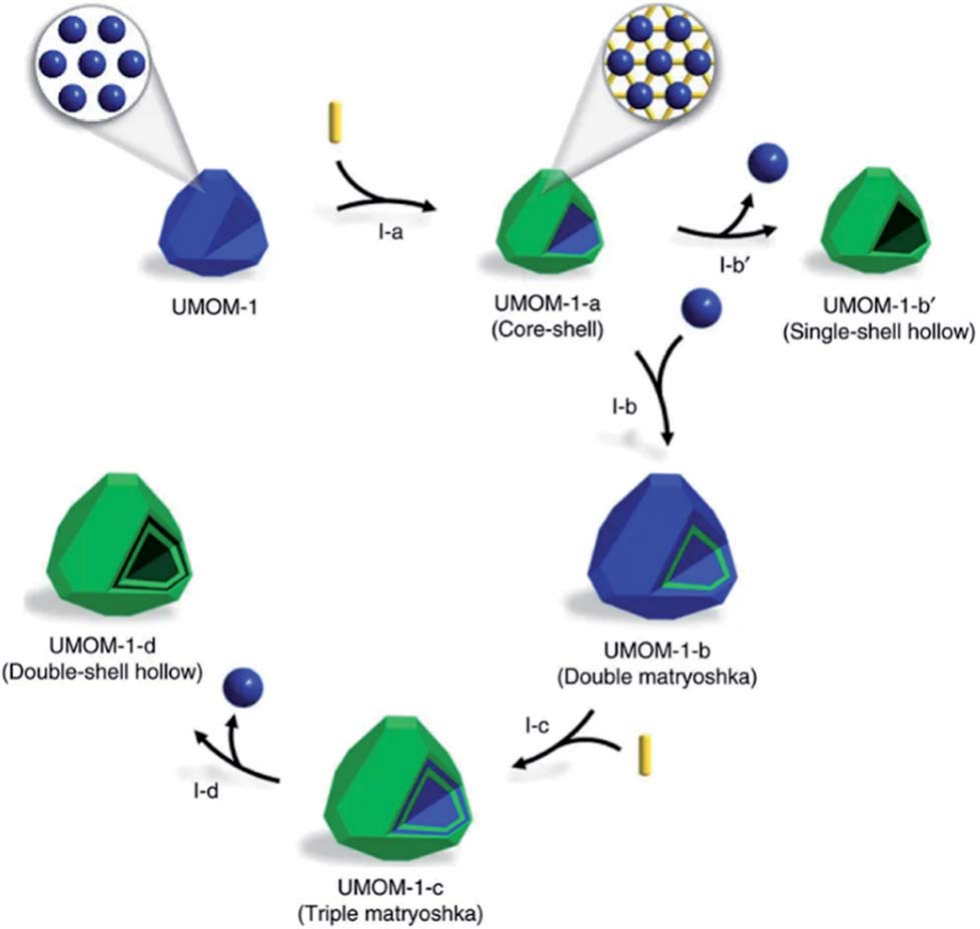
A schematic illustration of the synthesis of a double-shell HoMS–MOF through an interfacial reaction. Stage I-a: MOF@MOP (core–shell) by partial postsynthetic linker insertion from MOP. Stage I-b′: hollow MOF (single-shell hollow) by an etching process. Stage I-b: MOP@MOF@MOP (double matryoshka) by the epitaxial growth of MOP on the surface of MOF@MOP. Stage I-c: MOF@MOP@MOF@MOP (triple matryoshka) by partial post-synthetic linker insertion. Stage I-d: HoMS–MOF (double-shell hollow) by an etching process. The blue sphere represents MOP, the yellow rod represents the linker, and the green polyhedron represents the MOF. Reproduced with permission.^69^ Copyright 2017. The Authors. Published by Springer Nature.

Similar to the shell-by-shell formation, fabricating the alternate MOF@MOP (MOP = metal–organic polyhedra) structure can also be used to construct HoMS–MOFs.^68^ For instance, Choe's group reported the synthetic strategy to prepare multi-shelled hollow MOFs *via* a sequential interfacial reaction using MOP as the precursor. The cuboctahedron MOP (cuo-MOP) was selected as the starting material, it transformed into ubt-MOF by connecting adjacent precursors with dabco (1,4-diazabicyclooctanetriethylenediamine) linkers. The linker insertion reaction took place on the surface of the MOP, and the as-formed MOF layers prevented the linkers from penetration and prohibited further reaction with the inner part. Therefore, a core–shell structure of MOP@MOF is formed. Then another layer of MOP can be coated on the surface of MOF through epitaxial growth. After another surficial insertion reaction, the outermost layer of MOP converted into MOF. This process can be repeated several times and a multi-layered matryoshka-like core–shell structure (denoted as MOF@MOP@MOF@MOP) was formed because MOP was more vulnerable to chemical etching than MOF. The MOP layers can be removed under a proper etching condition, while the MOF layers remained. Thus, a HoMS–MOF was achieved (Fig. 6).^69^

**Fig. 7:**
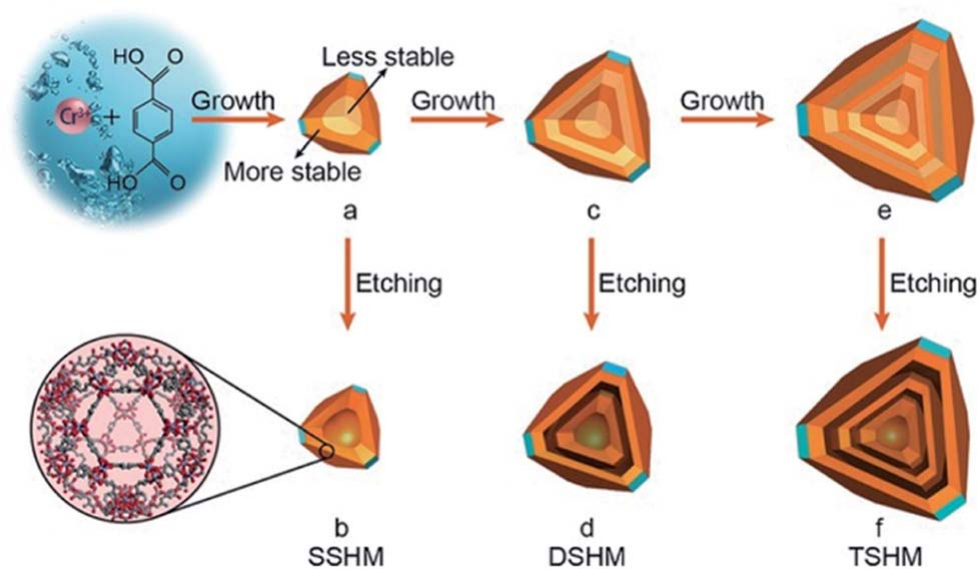
A schematic diagram of the fabrication of single-, double-, and triple-shelled hollow MIL101(Cr). (a) MIL101(Cr) with a more stable outer layer and a less stable inner core. (b) A single-shelled hollow MOF by the selective etching of product (a) with acetic acid. (c) The epitaxial growth of product (a). (d) Double-shelled hollow MOF *via* the etching of product (c). (e) The epitaxial growth of product (c). (f) Triple-shelled hollow MOF by the etching of product (e). The layers with a dark color represent the more stable part and the layers with a light color represent the less stable part. Reproduced with permission.^74^ Copyright 2017, Wiley-VCH.

In contrast to the selective etching of different MOFs, the fabrication of hollow MOFs starting with a single composition precursor is quite constructive,^70–72^ after determining that the inhomogeneity in the MOF is due to the unevenly distributed defects. Normally, the core of the as-synthesized materials has more defects, because of the rapid nucleation and growth at the initial stage^73^ and the defect-rich part was less stable than the well-crystallized part. The MOF crystal was in fact a core–shell structure that is expressed as less stable (LS)@more stable (MS). Under proper chemical etching conditions, the inner core can be removed while the outer shell remained, giving rise to a hollow structure. Impressively, if the shell-by-shell crystal growth was repeated several times, multi-layered core–shell labelled as LS@MS@LS@MS@LS@MS materials can be achieved. After etching away the LS part, multi-shelled MOFs can be obtained. Huo *et al.*^74^ chose MIL-101(Cr) as an example. After putting pre-synthesized LS@MS MIL-101 crystals into the mother solution for another hydrothermal reaction, the additional layer of MIL-101 can be epitaxially grown on the former truncated octahedral particles. The newly formed MOF layer also had a LS@MS structure. Subsequently, acetic acid was added to preferentially etch away the LS part, creating a hollow interior cavity. Additionally, the thickness of each shell can be tuned by controlling the crystal growth time and the cavity space varied with the different etching times. This rational strategy can effectively prepare HoMS–MOFs with single-crystalline shells (Fig. 7). HoMS–MIL101 has shown a better conversion rate and higher selectivity towards benzaldehyde for the styrene oxidation reaction compared with solid or less-shelled counterparts. The phase, morphology and catalytic performance remained unchanged after five reusing cycles. The structural advantages of the HoMSs provide more exposed active sites, which can improve the adsorption of the reactants. Following a similar shell-by-shell growth and subsequent etching method, other kinds of HoMS–MOFs can be fabricated as well.^75,76^ From the study, researchers discovered that HoMS–MOFs not only had a better adsorption capacity but also had a much slower release in the desorption isotherms, suggesting promise in applications favouring slow guest release, like drug delivery.

## 5. Outlook and conclusions

Both MOF materials and HoMSs possess diverse intrinsic physical and chemical characteristics that are interesting from both fundamental and practical perspectives. The combination of these two concepts can give both additional properties, which has shown its advantages in catalysis, energy conversion & storage and adsorption. However, it should be noted that this emerging topic still needs special attention in both the synthetic methodology and applications, and plenty of avenues still remain to be explored, including: (1) preparing HoMSs with more complex structures and compositions with various MOF precursors. For example, building up heterogeneous inorganic HoMSs is quite attractive. Great efforts should be made to contribute to putting this proof-of-concept into practice by using heterogeneous MOFs. For instance, to build heterogeneous MOFs with different metal elements inside out, and then to transfer them under proper conditions will give rise to heterogeneous HoMSs with different compositions in each shell. The hetero-HoMSs can successively absorb sunlight with different wavelengths in different shells from outside to inside (sequential solar-light-harvesting), broadening the absorption range of solar spectrum and enhancing the utilization of light.^77^ (2) For HoMS–MOFs, besides the shell-by-shell approach, there is still an urgent need for new methods. For example, we can predict that the solid HoMS could also be considered as a precursor and a template to direct the HoMS–MOF formation. Decades of study in HoMSs has provided a huge number of HoMSs with various compositions, which can all be adopted for the MOF synthesis. Depending on the composition of HoMS (including the homo and heterogeneous composition inside–out), the composition in the HoMS–MOF can be well tuned. This strategy opens up a new path for MOF synthesis especially structure control, providing new insights into how to utilize solid matters with specific morphologies and architectures as metal sources to realize certain fabrications. (3) Furthermore, one could also be interested in fabricating the HoMS & MOF hybrid, which means both the inorganic metal oxide (it can also be sulphide, *etc.*) and the MOF exist in different shells of a single HoMS particle. In this case, this hybrid system may be beneficial for the shape-selective catalysis and cascade reaction. Thus, it can give full play to the exact temporal–spatial control, which will provide a new catalytic schematic approach for industrial synthesis. (4) Last but not least, we would like to highlight another possibility for MOF-decorated HoMSs. The last few years have witnessed a boom in the utilization of MOFs for the preparation of single atom catalysts (SAC) to achieve large loading amounts and high dispersion of atomically active sites.^78–80^ It is reasonable to believe that loading SAs on HoMSs can create a cooperative activation from both the site-specific single atoms and multiple hollowness.

In summary, besides performing as a star material for various applications, MOFs can also bring lots of foundations for the precise synthesis of HoMSs, such as various morphologies, relatively lower metal element gradients, ordered porous structures and topological ordered metallic atom arrangements. In reverse, HoMSs can provide MOFs with enhanced mass transport and effective surface exposure, especially temporal–spatial ordering properties. The combination of MOFs and HoMSs is promising because it can not only enhance the properties of the materials but also can endow them with new functions. Although there are still many difficulties challenging us, researchers across the world have flocked to this research hotspot. Many more breakthroughs and much progress in both synthesis and applications can be expected in the near future. Hopefully, the insight gained from this perspective article will aid in the rational design and synthesis of other multi-shelled hollow structures and the further expansion of their applications.

## Conflicts of interest

The authors declare no competing interests.
